# An observational prospective cohort study of the epidemiology of hospitalized patients with acute febrile illness in Indonesia

**DOI:** 10.1371/journal.pntd.0007927

**Published:** 2020-01-10

**Authors:** Muhammad Hussein Gasem, Herman Kosasih, Emiliana Tjitra, Bachti Alisjahbana, Muhammad Karyana, Dewi Lokida, Aaron Neal, C. Jason Liang, Abu Tholib Aman, Mansyur Arif, Pratiwi Sudarmono, Suharto, Tuti Parwati Merati, Vivi Lisdawati, Siswanto, Sophia Siddiqui, H. Clifford Lane, for INA-RESPOND

**Affiliations:** 1 Faculty of Medicine, Universitas Diponegoro, Semarang, Indonesia; 2 Indonesia Research Partnership on Infectious Disease (INA-RESPOND), Jakarta, Indonesia; 3 National Institute of Health Research and Development, Ministry of Health, Republic of Indonesia, Jakarta, Indonesia; 4 Faculty of Medicine, Universitas Padjadjaran, Sumedang, Indonesia; 5 Department of Clinical Pathology, Tangerang District Hospital, Tangerang, Indonesia; 6 National Institute of Allergy and Infectious Diseases, National Institutes of Health, Bethesda, MD, United States of America; 7 Faculty of Medicine, Universitas Gadjah Mada, Yogyakarta, Indonesia; 8 Faculty of Medicine, Universitas Hasanudin, Makassar, Indonesia; 9 Faculty of Medicine, Universitas Indonesia, Jakarta, Indonesia; 10 Faculty of Medicine, Universitas Airlangga, Surabaya, Indonesia; 11 Faculty of Medicine, Universitas Udayana, Denpasar, Indonesia; 12 Sulianti Saroso, Infectious Disease Hospital, Jakarta, Indonesia; International Vaccine Institute, REPUBLIC OF KOREA

## Abstract

**Background:**

The epidemiology of acute febrile illness, a common cause of hospitalization in Indonesia, has not been systematically studied.

**Methodology/Principal findings:**

This prospective observational study enrolled febrile patients (temperature ≥38°C) aged ≥1 year from July 2013 until June 2016 at eight government referral teaching hospitals in seven provincial capitals in Indonesia. Patients were managed according to the hospital standard-of-care (SOC), and blood samples were drawn for molecular and serological assays. Clinical data, laboratory results, and specimens for additional tests were collected at enrollment, days 14–28, and at three months. Regular follow-up visits were then scheduled for every three months either until symptoms resolved or until one year. In total, this study included 1,486 adult and pediatric patients presenting with multi-organ (768, 51.7%), gastrointestinal (497, 33.0%), respiratory (114, 7.7%), constitutional (62, 4.2%), skin and soft-tissue (24, 1.6%), central nervous system (17, 1.1%), or genitourinary (4, 0.3%) manifestations. Microbiological diagnoses were found in 1,003/1,486 (67.5%) participants, of which 351/1,003 (35.0%) were not diagnosed during hospitalization using SOC diagnostic tests. Missed diagnoses included all cases caused by *Rickettsia spp*., chikungunya, influenza, and Seoul virus. The most common etiologic agents identified were dengue virus (467, 46.6%), *Salmonella spp*. (103, 10.3%), and *Rickettsia spp*. (103, 10.3%). The overall mortality was 89 (5.9%).

**Conclusions/Significance:**

Febrile illness in Indonesia has various microbiologic etiologies and substantial overall mortality. Diagnostic limitations and lack of epidemiologic data resulted in potentially treatable, and at times fatal, diseases being missed.

## Author summary

In tropical countries like Indonesia, fever due to infectious disease is the most common reason for hospitalization. However, diagnoses are mostly unconfirmed, as diagnostic tests are not available or are not performed due to budget constraints. Consequently, many patients are only treated based on clinical syndromes. To gain a better understanding of the epidemiology of acute fever in Indonesia, we conducted a study at eight hospitals in the seven largest cities from 2013–2016. We enrolled 1,486 subjects aged ≥1 year with acute fever ≥38°C. Blood cultures were mandatory for all subjects, while cultures of other biological specimens, microscopic examinations, and rapid tests for specific pathogens were based on clinical judgment and availability. Retrospectively, we performed molecular and serological testing for a panel of bacterial and viral pathogens for systemic, respiratory, and diarrheal diseases. We found six pathogens to be the most prevalent: dengue virus (47%), *Salmonella* Typhi*/*Paratyphi (10%), *Rickettsia typhi* (10%), influenza virus (7%), *Leptospira spp*. (5%), and chikungunya virus (4%). *Rickettsia typhi*, influenza, and chikungunya had not been considered in the differential diagnosis of any subject at the hospitals. Thus, multiple pathogens were associated with acute febrile illnesses, and a subset of treatable cases were missed. This may have resulted in increased overall mortality.

## Introduction

Indonesia is a geographically diverse archipelago with a population of 240 million [1]. Fever is a common presenting symptom among hospitalized patients, and inadequate data on fever etiologies in the country [2,3] has significant implications for clinical management, antibiotic usage, and public health interventions.

The current standard-of-care (SOC) is largely based on clinical judgment, with dengue and typhoid fever being the most frequent default clinical diagnoses. Diagnostic blood cultures are not routinely performed. In Indonesia and other developing countries, most epidemiological studies of diseases in hospitalized patients presenting with fever have largely focused on one or a few specific pathogens [4–13]. This leaves a substantial proportion of cases undiagnosed. The objectives of the present study were to identify the etiologies of febrile illnesses and to evaluate the clinical manifestations, microbiology, and mortality for patients with new onset fevers hospitalized across eight reference hospitals in Indonesia.

## Methods

### Ethics statement

Ethical approvals were received from the institutional review boards of Dr. Soetomo Hospital, Faculty of Medicine, Universitas Indonesia, and the National Institute of Health Research and Development (NIHRD), Ministry of Health, Indonesia. The study was conducted in accordance with the ethical principles stated in the Declaration of Helsinki. Patients were eligible for participation if they or a legal guardian/representative provided consent following an explanation of the study objectives and procedures in Indonesian. Assent was obtained from children ≥13 years old or who were old enough to understand the proposed research. Investigators read and explained the informed consent in Indonesian to illiterate participants who then either signed the consent or gave a thumb-print alongside the signatures of witnesses.

### Study sites and approvals

This longitudinal study was conducted by the Indonesia Research Partnership on Infectious Disease (INA-RESPOND) [14] and enrolled participants at eight government referral teaching hospitals in seven provincial capitals in Indonesia. These hospitals are tertiary health care facilities equipped with 700+ beds (with the exception of Sulianti Saroso Infectious Disease Hospital which has only 126 beds), diagnostic laboratories, and specialty physicians and departments. Diagnostic laboratories are capable of performing routine hematological, biochemical, microbiological, molecular, and serological testing. More advanced molecular and serological tests are infrequently performed, as noted above. Catchment populations are representative of urban, rural, or a combination of both regions, are mostly of the low- to middle-income socioeconomic range, and are dependent on the National Insurance System for healthcare.

### Participants and study procedures

Eligibility criteria included being ≥1 year of age, having an axillary body temperature of ≥38°C, and being hospitalized within the past 24 hours. Patients were excluded from participation if they had subjective fever for more than two weeks or were hospitalized in the preceding three months (S1 Fig). Participants were enrolled between July 2013 and June 2016, with an original target of 100 adult and 100 pediatric patients per site over two years. The study enrollment was closed at 1,486 participants due to the introduction of a new health insurance system that reduced the number of acute fever patients presenting at the tertiary care hospitals that were participating study sites.

At enrollment, demographic, clinical, and laboratory data were collected through interviews and from medical records. Peripheral blood specimens were obtained from both arms of each participant for blood cultures at the hospitals and for preservation in a biorepository. Other biological specimens (nasal swabs, sputum, feces, urine) were obtained as requested by the physician in charge as part of SOC. SOC was based on available tests at the hospital and the clinical judgment of the physician in charge. SOC for laboratory tests typically included culture of sputum/urine, microscopic examination of sputum/urine/feces, complete blood count, rapid tests for anti-dengue IgM and IgG antibodies, dengue NS1 antigen, anti-*Salmonella* Typhi IgM antibodies, and in two hospitals, anti-*Leptospira spp*. IgM antibodies. Any biological specimens remaining after SOC testing were stored for future study.

Blood specimens, clinical data (when available), and/or outcomes were also collected at baseline, 14–28 days, and three months after enrollment. After the third visit, participants with symptoms persisting from the initial presentation were followed every three months until resolution of symptoms or up to 12 months post-enrollment. For all study visits after the baseline visit, participants returned to the same hospital where enrollment occurred. If a participant was unable to return to the hospital for follow-up, a home visit was conducted to obtain the necessary study data and specimens. Final diagnoses were based on hospital and INA-RESPOND Laboratory findings.

### Definition of clinical presentations

Based on the clinical signs and symptoms presented at the time of enrollment, participants were categorized as having constitutional, central nervous system (CNS), respiratory, gastrointestinal, genitourinary, and/or skin and soft-tissue clinical manifestations (S1 Table). When a non-infectious disease process, such as Systemic Lupus Erythematosus (SLE), was diagnosed the patient was categorized as having a non-infectious disease.

### Laboratory testing and pathogen identification

Suspected pathogens were identified by the hospital laboratories and/or the INA-RESPOND Laboratory at Tangerang District Hospital. At the hospitals, blood cultures were performed using BacTek or Vitek systems that had been widely used and validated. A variety of rapid diagnostic tests from different manufacturers were used to detect dengue virus and *Salmonella* Typhi infections. All results from these rapid tests were retrospectively confirmed or rejected using molecular and serological tests conducted at the INA-RESPOND Laboratory. Probable etiologies were considered confirmed when clear evidence of a pathogens was observed by culturing, microscopy, molecular testing, and/or serological testing (S2 Table). Due to the complexity of the pathogens, their various clinical manifestations, and the sample types and timepoints available in this study, etiology confirmation was determined on a case-by-case basis by rigorously evaluating all available clinical and laboratory data rather than by following strict confirmation criteria, such as requiring both PCR and IgM ELISA positivity for a given pathogen. The INA-RESPOND Laboratory performed molecular and serological assays that could not be performed at the hospitals, using the testing algorithm shown in S2 Fig. Briefly, since dengue is highly prevalent in Indonesia and results in clinical presentations similar to other infectious diseases all subjects were first screened for acute dengue infection using RT-PCR, NS1 antigen ELISA, and IgM and IgG antibody tests. Dengue-negative subjects with negative blood cultures and/or other SOC gold-standard test results were then screened for four additional pathogens: *Salmonella enterica*, *Rickettsia spp*., *Leptospira spp*., and chikungunya virus. This panel was selected based on the generally high prevalence of these pathogens in the tropics, particularly in Indonesia [3,10,11].

Blood specimens from subjects with unknown etiologies following the investigations described above, were tested as follows: a) in all pediatric subjects, blood specimens were tested for HHV-6 using molecular techniques; b) in subjects with respiratory infections, blood specimens were tested serologically for influenza A and B; c) in pediatric subjects with respiratory infections, blood specimens were tested serologically for RSV; d) in subjects with morbilliform rash, blood specimens were tested molecularly and serologically for measles; e) in subjects suspected to have rubella, blood specimens were tested serologically for rubella; and f) in subjects with jaundice, elevated liver enzymes, or renal insufficiency, blood specimens were tested molecularly and serologically for hantavirus. Lastly, all subjects were screened for HIV using two rapid serological tests, a rapid fourth-generation ELISA, and RT-PCR.

### Adjudication process for multiple pathogens

The etiology of fever in a participant was attributed to multiple pathogens only when there was clear evidence of infection with multiple pathogens. In these cases, each pathogen had to be identified by either positive culture (blood or other sterile fluid), positive PCR from blood, sero-conversion or four-fold increase of IgM/IgG, positive microscopic examination of feces or sputum, positive sputum culture, or positive PCR from culture. Additionally, the participant had to have a clinical presentations that was compatible with the identified pathogens.

### Data collection, storage and statistical analysis

Data were recorded on paper case report forms and entered in duplicate into OpenClinica (OpenClinica, LLC) by clinical research staff. Descriptive statistics were calculated using Stata 13 (StataCorp, LLC). Proportion/frequency for participant characteristics, etiologies of febrile illnesses, clinical manifestations, and mortality were determined. Age groups were created to be consistent with the age groups typically defined in national census programs in order to provide insight into the impact of disease on key groups (i.e. school-aged, workforce, elderly, etc.). When data were presented according to study site, the Sulianti Saroso Infectious Disease Hospital and Cipto Mangunkusumo Hospital (both in Jakarta) were treated as a single site. Missing data were treated as missing. Other variations in “n” were due to withdrawals, early termination, and lost to follow-up (S1 Fig).

## Results

### Baseline characteristics

A total of 5,213 patients were screened, of whom 1,492 consented and were enrolled. Six participants were excluded from data analysis because they did not meet the inclusion/exclusion criteria, resulting in a total of 1,486 participants. The participants ranged in age from 1 to 98 years, with a median of 20.8 years; of these, 623/1,486 (41.9%) were pediatric patients (S2 Table). The study completion rate was 89.3% (1,327/1,486), with 1,217 (81.8%) participants being followed-up for 3 months and 21 (1.4%) participants for 6–12 months. Eighty-nine (5.9%) participants died during the study. The remaining 159 (10.7%) participants either withdrew from the study or were lost to follow-up (S1 Fig).

Participant characteristics are shown in Table 1. The median duration of fever prior to hospitalization was four days, and the median duration of hospitalization was six days (range of 1–55 days). An underlying pre-existing condition was present in 506/1,455 (34.8%) participants. The presence of pre-existing conditions was higher in patients aged >65 years (85.7%) and 45–65 years (58.1%) compared to those <5 years (39.2%), 6–18 years (24.8%), and 18–25 years (24.9%). Pre-existing conditions consisted of malnutrition, anemia, and congenital malformation in participants aged <5 years; malnutrition, tuberculosis, and CNS diseases in participants aged 5–18 years; tuberculosis, HIV, and anemia in participants aged 18–45 years; and metabolic, circulatory, and respiratory diseases in participants aged ≥45 years.

**Table 1:** Patient characteristics and syndromes.

Patients Characteristics		n = 1,486	
Male patients, n (%)		830 (55.9%)	
Median age, years (range, IQR)		20.8 (1.0–98.0, 9.1–37.9)	
Mean age, years (SD)		25.6 (19.7)	
Age group			
≤5 years, n (%)		210 (14.1%)	
6–18 years, n (%)		413 (27.8%)	
>18 years, n (%)		863 (58.1%)	
Underlying disease(s), n = 1,455, n (%)		506 (34.8%)	
Days of onset before hospitalization, n = 1,467, median (range, IQR)		4 (0–35, 3–6)	
Self-reported antibiotic use prior to hospitalization			
Yes, n (%)		394 (26.5%)	
No, n (%)		851 (57.3%)	
Unsure, n (%)		241 (16.2%)	
Days hospitalized, n = 1,476, median (range, IQR)		6 (1–55, 5–8)	
Days in ICU, n = 26, median (range, IQR)		5 (1–48, 4–8.3)	
Syndromes, n (%)			
Constitutional	62 (4.2)	Skin and Soft Tissue	24 (1.6)
Respiratory	114 (7.7)	Central Nervous System	17 (1.1)
Gastrointestinal	497 (33.4)	Multi-organ	768 (51.7)
Genitourinary	4 (0.3)		

Notes: Variation in number of participants (n) was due to missing data.

### Clinical manifestations

The majority of patients (51.7%) had multi-organ clinical manifestations. The most common symptoms accompanying fever were nausea (920, 61.9%), headache (593, 39.9%), and anorexia (496, 33.4%). Among patients with single-organ clinical manifestations, gastrointestinal (33.4%) and respiratory (7.7%) syndromes were most common. Antibiotics were received by 394 (26.5%) participants prior to hospitalization, with 280 recalling the specific antibiotics received. The clinical profiles of participants are shown in Table 1. Most participants presented with stable vital signs and hematological parameters. Twenty-six participants (1.7%) were admitted to an intensive care unit of whom 10 (38.5%) died.

### Microbiology

**Fig 1:**
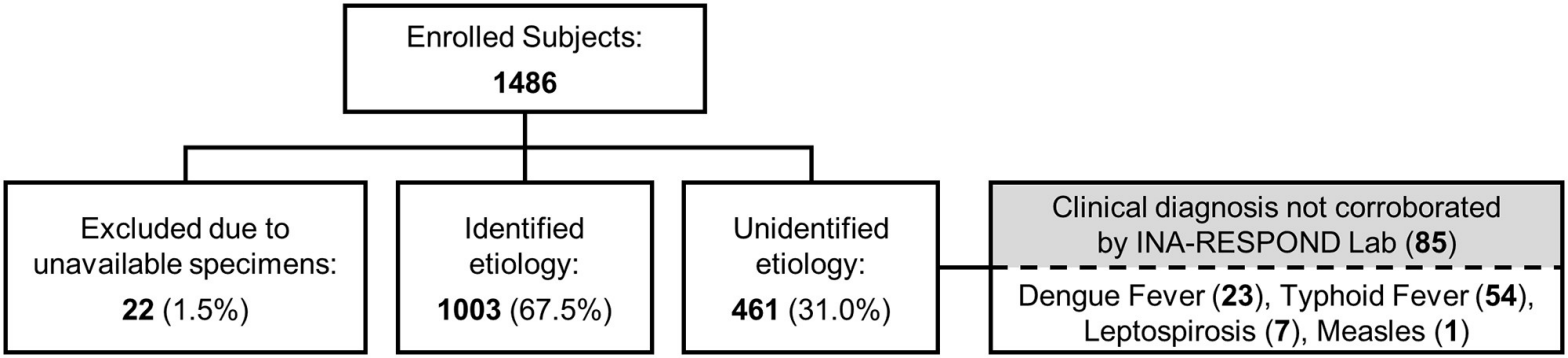
Confirmed etiologies in 1,003 participants.

Microbial etiologies were eventually identified in 1,003 (67.5%) participants. Identifications in 652 (43.9%) were made by SOC tests at the hospitals, while identifications in the remaining 351 (23.6%) were made by the INA-RESPOND Laboratory (Fig 1) using methods described in S3 Table. Diagnostic discrepancies between hospital SOC and INA-RESPOND laboratory diagnoses are described in S4 Table.

**Fig 2:**
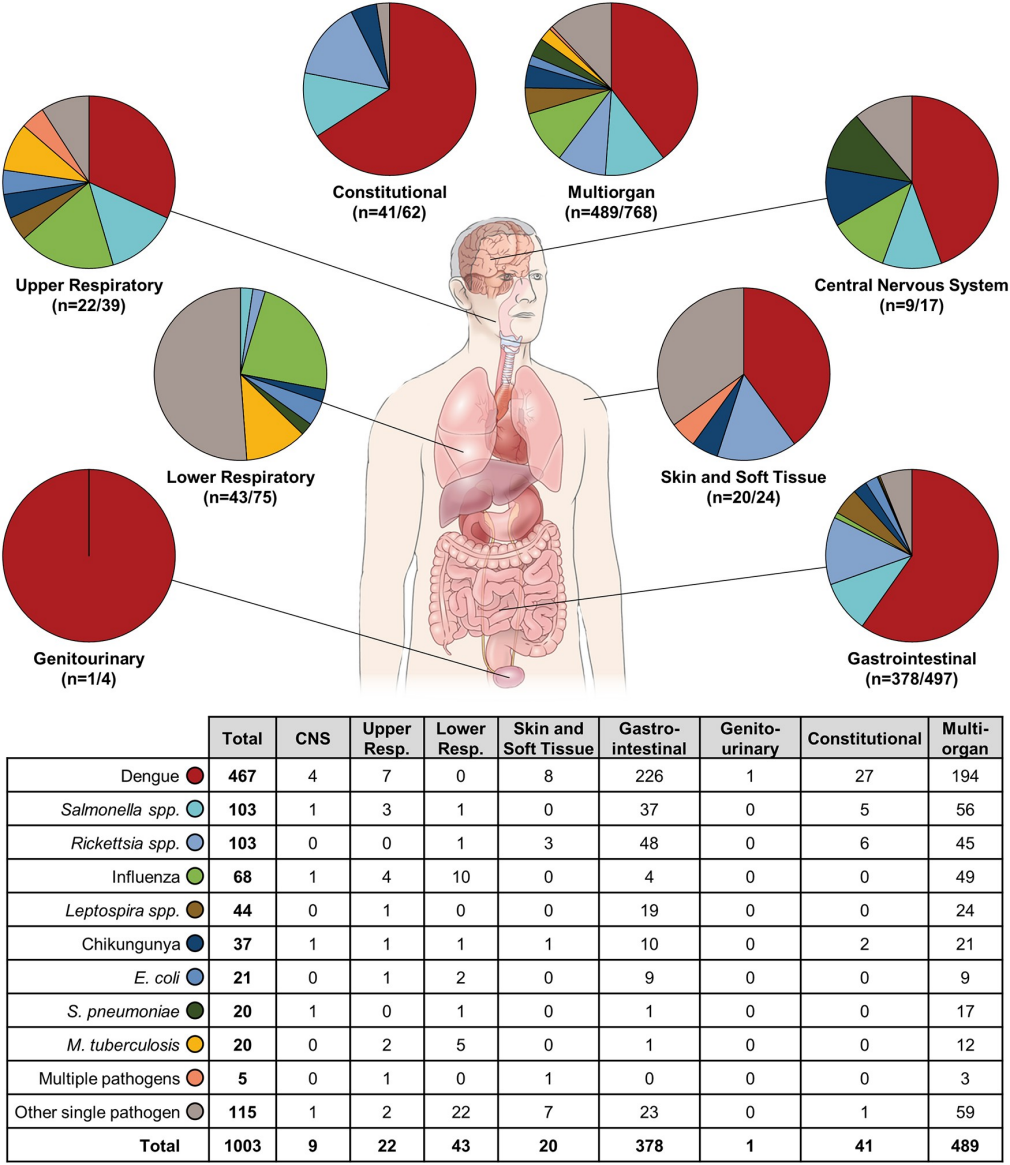
Representation of the most common microbiologic etiologies by organ system.

**Fig 3:**
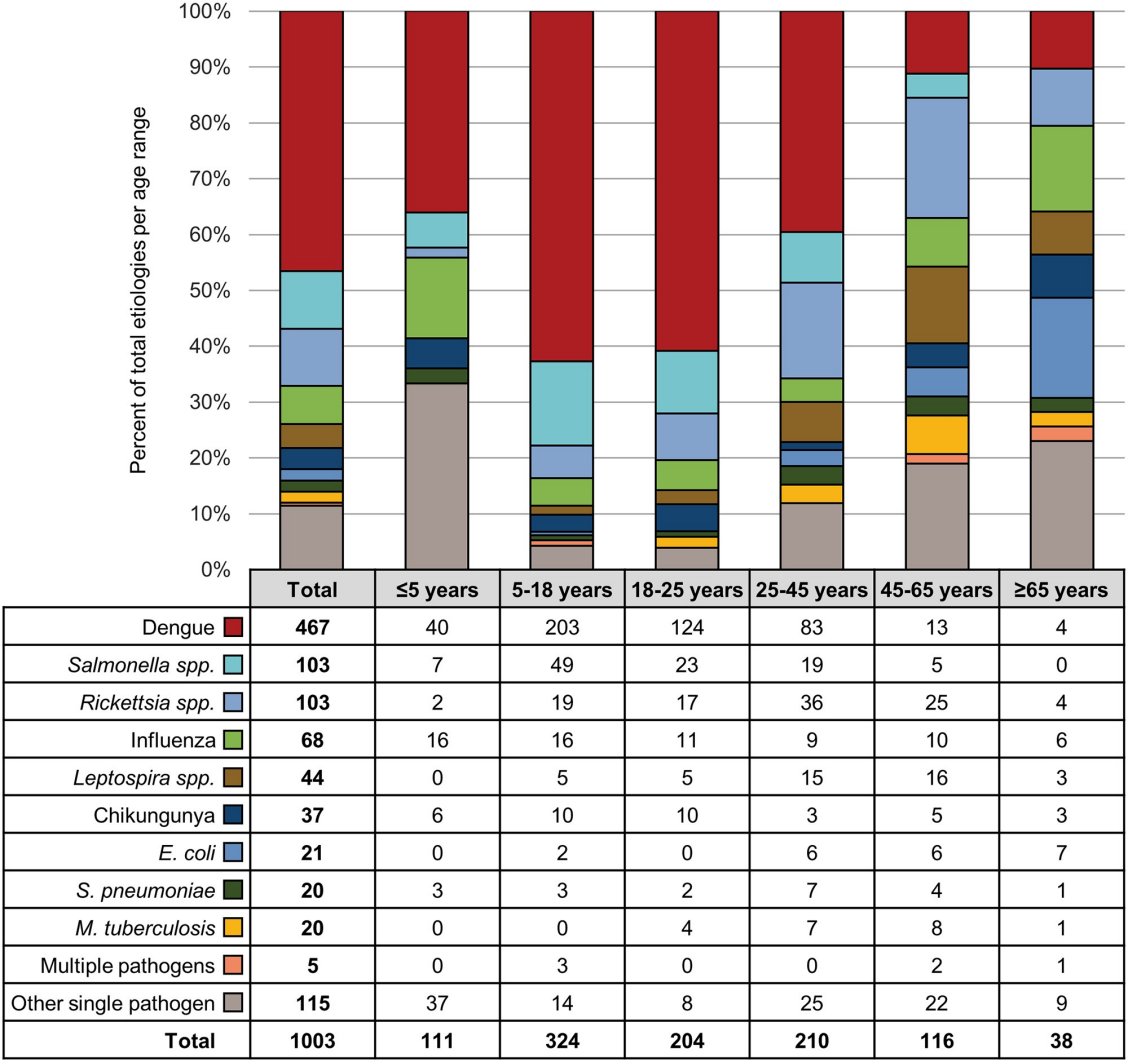
Representation of the most common microbiologic etiologies by age group.

**Fig 4:**
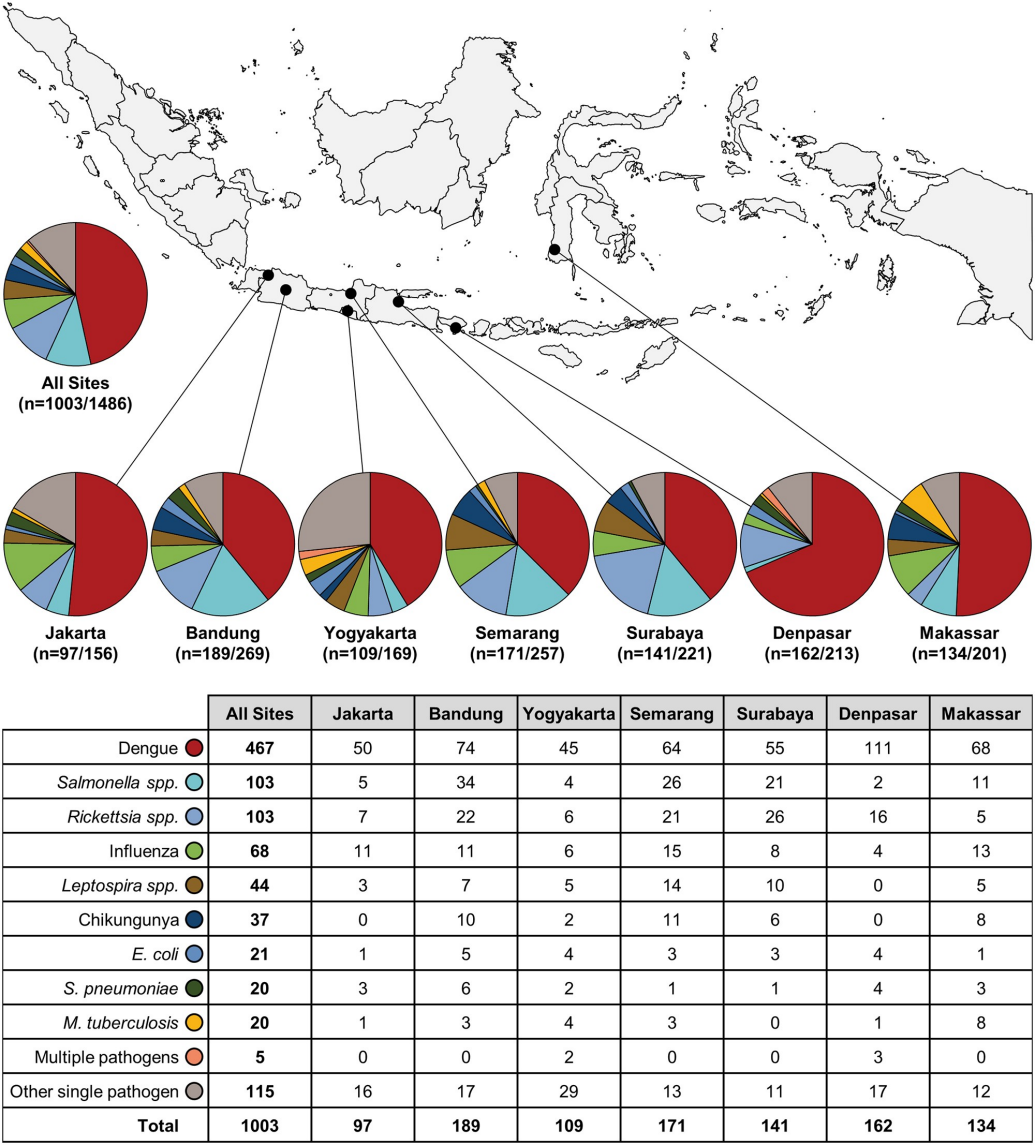
Representation of the most common microbiologic etiologies by study site.

Pathogens were more frequently identified in participants presenting with symptoms specific to a single organ or system compared to those presenting with multi-organ symptoms (71.6% vs. 63.7%) (Fig 2). The most common etiology across all age groups and sites, regardless of organ system manifestations, was dengue (467, 31.5%), followed by *Salmonella enterica* (103, 6.9%) and *Rickettsia spp*. (103, 6.9%) (Figs 3 and 4). Pathogens associated with different organ systems are listed and described in Fig 2 and S5 Table.

Three infectious etiologies not captured by SOC testing or documented as a discharge diagnosis were *Rickettsia spp*. (*R*. *typhi* and *R*. *felis*), chikungunya, and influenza. These etiologies were identified solely by the INA-RESPOND Laboratory and were found across all age groups and at all eight study sites (Figs 3 and 4). *Rickettsia spp*. accounted for 103/1,003 (10.3%) of all confirmed cases and were often misdiagnosed as *Salmonella spp*. or dengue infections. The highest number of cases of rickettsiosis (36) was seen in the 25–45 year age group and accounted for 35.9% of all diagnoses of *Rickettsia spp*. Diagnoses of *Rickettsia spp*. were confirmed by PCR and serology, serology-only, or PCR-only in 63 (62.8%), 36 (35.0%), and 4 (3.9%) participants, respectively. Chikungunya virus was confirmed in 37/1,003 (3.7%) participants, and the majority of those cases (26, 70.3%) were among participants <25 years of age. Chikungunya virus was diagnosed by PCR and serology in 30 (81.1%) participants and by serology-only in 7 (18.9%) participants. Influenza virus was confirmed in 68 (6.8%) cases by either serology-only (61, 89.7%) or PCR and serology (7, 10.3%) (S6 Table). Three other pathogens not routinely considered but identified by the INA-RESPOND Laboratory were Seoul virus (2, 0.2%), HHV-6 (9, 0.9%), and RSV (11, 1.1%) (S4 Table).

### Mortality

**Fig 5:**
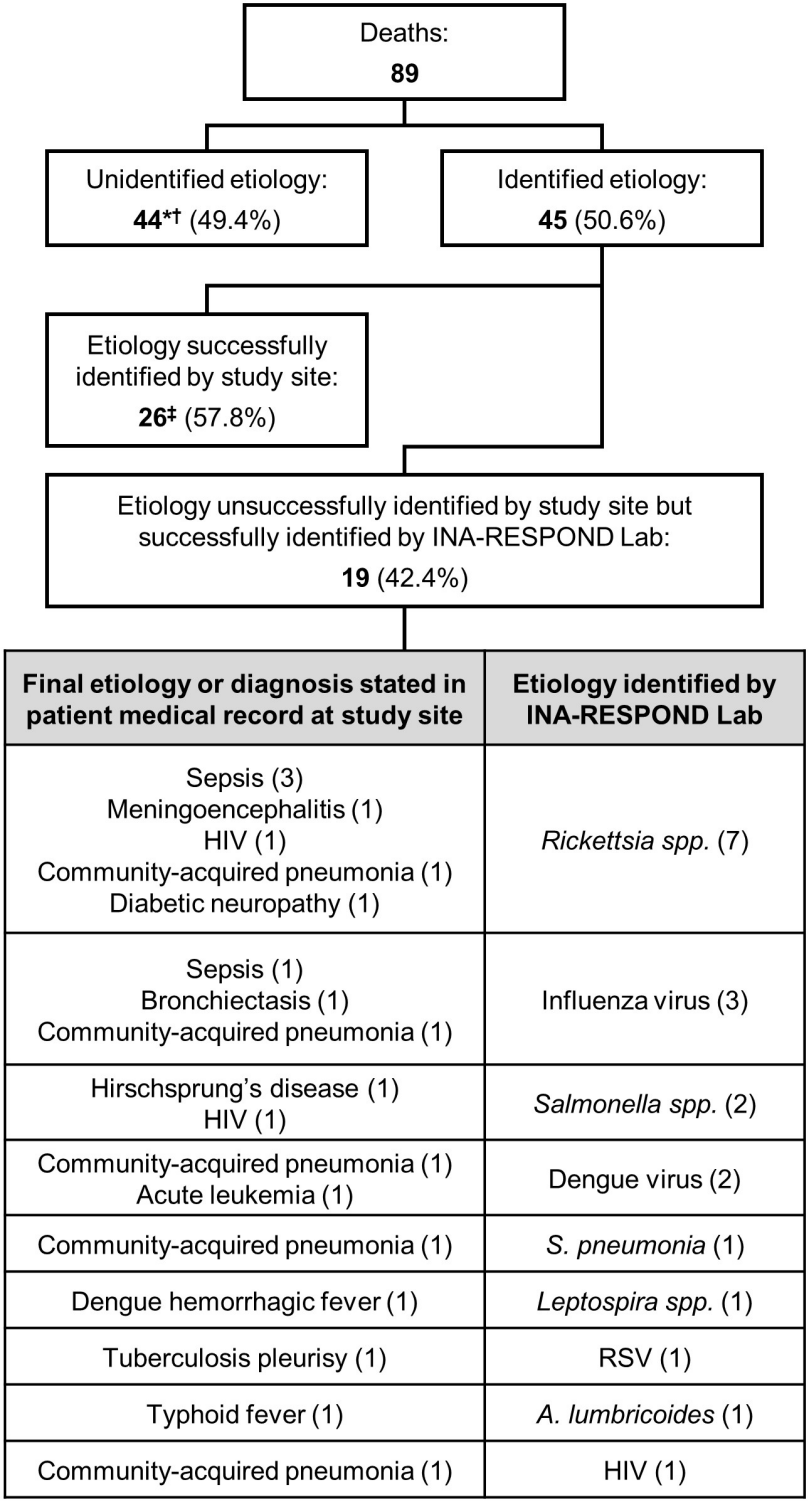
Microbiologic etiologies identified in 19 cases that were discordant with the final diagnosis listed in the medical record. *For 2 patients, the INA-RESPOND Lab was unable to identify etiologies but was able to reject the etiologies identified by the sites. ^†^Etiologies could not be determined for 2 patients since they died after enrollment but before sample collection. ^‡^For 1 patient with a co-infection, *M*. *catarrhalis* was successfully identified by the site, but influenza and *S*. *pneumoniae* were missed.

89 participants (72 adult and 17 pediatric) died, yielding an overall mortality of 5.9%. The highest mortality was seen in the 45–65 year age group (31/203, 15.3%) followed by the 25–45 year age group (25/327, 7.6%) **(**S7 Table). The three most common microbiologic etiologies identified in the fatal cases were *M*. *tuberculosis* (8/89, 8.9%), *R*. *typhi* (7/89, 7.8%), and *Salmonella spp*. (5/89, 5.6%). These three diagnoses were found to have mortality rates of 40.0% (8/20), 6.8% (7/103), and 4.9% (5/103), respectively. Etiologies in 19 participants were not diagnosed by the study site but were identified by the INA-RESPOND Laboratory, with the most common being *Rickettsia spp*. (7/19, 36.8%) and influenza (3/19, 15.8%) (Fig 5). A microbiologic etiology was not identified in 44 deaths (49.4%). The proportion of participants with underlying pre-existing conditions was higher in the deceased group compared to those who survived (80.2% vs. 31.9%).

HIV infection was identified in 46/1,486 (3.1%) participants, 6 (13.0%) of whom were newly identified during INA-RESPOND Laboratory testing. The mortality rate for these cases was 37%. In addition to HIV, other underlying conditions among those who died included diabetes mellitus, pulmonary tuberculosis, anemia, congestive heart disease, chronic kidney disease, cancer, and SLE (45.6%). The most common clinical cause of death was septic shock, followed by respiratory failure (S7 Table).

## Discussion

This study is the most comprehensive research to-date in Indonesia on identifying the etiologies of febrile illnesses leading to hospitalization. Using a combination of current SOC diagnostic tests and additional molecular and serological assays performed at the INA-RESPOND Laboratory, the probable causes of fever were successfully identified in 67.5% of participants. Unlike previous studies focusing on specific pathogens, the approach in this study allowed for the screening of a large number of pathogens. This led to a number of diagnoses of pathogens not included or considered in the original differential diagnoses. Since the pathogens were confirmed by molecular and/or serological assays, it is likely that they were the probable causes of fever. This study demonstrates that even commonly seen pathogens, such as dengue virus, may have various clinical presentations, meaning that diagnoses based solely on conventional clinical judgment can be misleading [15].

The rate of confirmed microbiological diagnoses in this study was 67.5%, which is higher than similar studies in southern Laos, Cambodia, Thailand, and Myanmar where pathogens were identified in 52% [6], 38% [8], 39% [9], and 48% [4] of cases, respectively. The increased rate of diagnoses in this study was likely due to a larger diagnostic panel, the utilization of acute and convalescent blood specimens, and the examination of other biological samples in addition to blood. In contrast to similar studies in the region, Japanese encephalitis, scrub typhus, spotted fever, and melioidosis were not identified in this study, although cases of murine typhus were identified. Since the sites included in this study were located mostly in provinces where malaria has been eradicated [16], *Plasmodium spp*. were only identified in two cases, both of which were imported.

The proportion of all cases confirmed as being due to dengue virus (31.4%) was similar to that in a study conducted in Vietnam [17], highlighting the importance of this pathogen and the need for better detection, prevention, and treatment strategies. As expected, dengue was less common in older subjects [18]. This likely reflects the development of protective immunity following lifelong exposure to multiple serotypes.

The INA-RESPOND Laboratory, which tested specimens by PCR and/or IgM and IgG ELISAs, confirmed only one-third of the *Salmonella spp*. cases diagnosed by rapid diagnostic tests at the hospitals. In most cases, other pathogens were detected. This highlights the importance of re-evaluating the performance of this rapid diagnostic test, especially in tropical settings where it is commonly used and often relied upon as the sole means for making a diagnosis.

A surprising finding was the presence of rickettsiosis in Indonesia. None of the laboratory-identified 103 cases of *Rickettsia spp*., including seven deceased participants, were clinically suspected as rickettsiosis. Although *Rickettsia typhi* has been reported among vectors in Indonesia [19], reports of human disease are extremely limited, and clinical relevance has not been thoroughly established [12]. Data regarding the prevalence of prior infection are rare and mostly based on serological results [20]. In other Asian countries, primarily India, Thailand, Laos, Cambodia, and Malaysia, rickettsioses have been reported as one of the major causes of fever [21,22]. In these high-prevalence countries, doxycycline is recommended to be given empirically [21–23]. Since the clinical manifestations of rickettsiosis often resemble those of salmonellosis, dengue infection, and leptospirosis, misdiagnosis of this infection is common and potentially fatal. Furthermore, false positive results from rapid diagnostic tests for *Salmonella spp*. were seen in 37% of *Rickettsia spp*. cases, complicating the accurate diagnosis of rickettsiosis in Indonesia.

The lack of diagnosis of influenza seen at hospitals is important, particularly given the high number of cases (68, 6.8% of all diagnoses) and observed fatality rate (4.3%). Most of these cases were diagnosed as non-specific upper respiratory tract infections or pneumonia. These findings have clear implications for hospital infection control, the role of vaccination, particularly in vulnerable populations, and surveillance for the emergence of novel influenza strains [24].

*Leptospira spp*. was the fourth most common pathogen observed in this study occurring in 44 (4.4%) of all diagnosed cases. It was often misdiagnosed clinically as dengue. Misdiagnosis was especially common when the clinical signs and symptoms were atypical. Leptospirosis is often overlooked in dengue endemic countries, and as with rickettsiosis, this can lead to severe complications or death if the administration of appropriate antibiotics is delayed [25,26]. In this study, a fatal case of leptospirosis was initially diagnosed as dengue and the participant never received antibiotics during hospitalization.

In addition to dengue, another common arbovirus present at all sites was chikungunya. This finding is consistent with a previous study in Indonesia demonstrating that cases of chikungunya are not limited to outbreaks but are also prevalent between outbreaks [27]. The identification of measles virus in a number of subjects, including adults with no antibodies during the acute illness, highlights the need for comprehensive vaccination in Indonesia. Though vaccines for influenza viruses [28], *Salmonella* typhi [29], and measles are available, an effective and safe vaccine for dengue is still needed. The most promising vaccine to-date (Dengvaxia), which is recommended by the WHO for high endemic areas [30], has been associated with severe post-vaccination infection in patients naïve for dengue virus prior to immunization [31].

Like other studies [32,33], the results of this study demonstrate that fatalities are most common in older individuals and in those with underlying comorbidities. Fatalities occurred mostly during hospitalization and were due to septic shock or respiratory failure. There were 19 patients who died from potentially treatable diseases that were identified only by the INA-RESPOND Laboratory (Fig 5). Appropriate and potentially life-saving treatments were not administered in these subjects.

The present study highlights the need for better and more widely implemented point-of-care rapid diagnostic tests. Rapid tests based on antigen detection are preferred over those based on antibody response, as patients can be diagnosed and treated more rapidly since antigen is detected earlier than IgM antibodies. However, the development and deployment of robust rapid antigen-based tests is challenging [34,35].

The routine inclusion of blood cultures in the diagnostic workup provided clear value in this study, especially for infections with *Salmonella enterica* and other bacteria associated with bacteremia, such as *Streptococcus pneumonia*, *Escherichia coli*, and *Klebsiella pneumonia*. However, the positive yield from cultures was low (5.9%) and was likely affected by the use of antibiotics prior to hospitalization among 26.5% of subjects. Blood culture is not part of SOC at hospitals in Indonesia, as it is expensive and not always covered by the national health insurance system.

This study has several limitations. First, enrollment was conducted in eight major hospitals in seven large cities on only three of the 15,705 islands of Indonesia [36], thus limiting the ability to generalize findings to the entire country. However, the hospitals included in the study were the top referral hospitals in the seven most densely populated provinces and cover 52% of the population of Indonesia [36], thus providing a reasonable representation of the population. Another limitation is the lack of systematic collection of specific specimens other than blood. This approach meant that only a subset of organ-specific samples was available for molecular diagnostic assays. A final limitation is that thorough causal testing was not conducted, which means that some of the organisms identified may not have been the causes of fever. Many potentially pathogenic organisms can also be found in healthy individuals, and in the case of serology, results may be reflective of a past infection with the identified organism. To reduce the possibility of false positive results, strict criteria were followed for molecular and serologic diagnoses, including high cut-offs and/or the need for increasing antibody titers (S3 Table).

This study is the most comprehensive study in Indonesia to-date identifying the causes of acute febrile illness requiring hospitalization. These data collected over three years span both rainy and dry seasons and provide the clearest picture yet of the infectious etiologies of fever in Indonesia. In this study, a combination of diagnostic tests was performed systematically at the hospitals (blood culture) and at the INA-RESPOND Laboratory (molecular and serological tests), allowing for the detection of a majority of pathogens associated with fever. This testing was greatly enhanced by the availability of convalescence specimens, which aided in the identification of pathogens that have short-lived or low-level circulating viremia/bacteremia and that typically require indirect detection through serology.

This study highlights the variety of microbiological etiologies of febrile illness in Indonesia, including six pathogens of particularly high prevalence: dengue virus, *Salmonella* Typhi, *Rickettsia typhi*, influenza virus, *Leptospira spp*., and chikungunya virus. Only cases of dengue virus infection were generally diagnosed accurately, which may be due to the widespread availability of IgM-based and antigen-based rapid diagnostic tests. The lack of diagnosis of all *Rickettsia typhi*, influenza, and chikungunya cases, in addition to 70% of the fatal cases, is especially concerning. *Rickettsia typhi* in particular is a potentially lethal but easily treatable pathogen, yet it was never considered as a diagnosis at any of the hospitals. Improved diagnoses for this and other pathogens may lead to more appropriate management and treatment of cases, leading to a reduction in overall mortality. Additionally, an improved understanding of disease prevalence through improved diagnoses may also aid public health authorities in conducting targeted pest control programs to reduce disease transmission in the community. Unfortunately, approximately 33% of participants in this study remained without a diagnosis, so further research should be performed to understand the full spectrum of febrile illness associated with hospitalization in this region of the world. Nonetheless, the identification of an etiology in 67.5% of cases is a marked improvement from the 40–50% observed in other studies in the region, and this further highlights the benefits of enhanced laboratory capacity to improve diagnosis and, by extension, the management of febrile illnesses.

## Supporting information

S1 Fig Subject screening, enrollment, and monitoring flowchart. (PDF)

S2 Fig Diagnostic laboratory algorithm. (PDF)

S1 Table Definitions for categorizing clinical manifestations. (PDF)

S2 Table Demography of the participants at all study sites. (PDF)

S3 Table Molecular and serology tests at the INA-RESPOND Laboratory. (PDF)

S4 Table Diagnostic discrepancies between clinical or hospital SOC testing and INA-RESPOND Laboratory diagnosis. (PDF)

S5 Table List of pathogens according to clinical syndromes. (PDF)

S6 Table Methods of pathogen confirmation. (PDF)

S7 Table Characteristics of deceased participants. (PDF)

S1 Checklist STROBE checklist. (DOC)

S1 Appendix (XLSX)
