# Correction: An observational prospective cohort study of the epidemiology of hospitalized patients with acute febrile illness in Indonesia

**DOI:** 10.1371/journal.pntd.0010530

**Published:** 2022-06-09

**Authors:** Muhammad Hussein Gasem, Herman Kosasih, Emiliana Tjitra, Bachti Alisjahbana, Muhammad Karyana, Dewi Lokida, Aaron Neal, C. Jason Liang, Abu Tholib Aman, Mansyur Arif, Pratiwi Sudarmono, Suharto, Tuti Parwati Merati, Vivi Lisdawati, Siswanto, Sophia Siddiqui, H. Clifford Lane, INA-RESPOND

The Funding statement is incorrect. The correct Funding statement is as follows: This project has been funded in whole or in part with MOH Indonesia and Federal funds from the National Institute of Allergy and Infectious Diseases, National Institutes of Health, under contract Nos. HHSN261200800001E and HHSN261201500003I. The content of this publication does not necessarily reflect the views or policies of the Department of Health and Human Services, nor does mention of trade names, commercial products, or organizations imply endorsement by the U.S. Government.
