# Supplementary figures and images for: An observational prospective cohort study of the epidemiology of hospitalized patients with acute febrile illness in Indonesia
Source: PLoS Negl Trop Dis. 2020 Jan 10;14(1):e0007927. doi: 10.1371/journal.pntd.0007927 (PMC6977771; doi:10.1371/journal.pntd.0007927)

**S1 Fig. Subject screening, enrollment, and monitoring flowchart.**

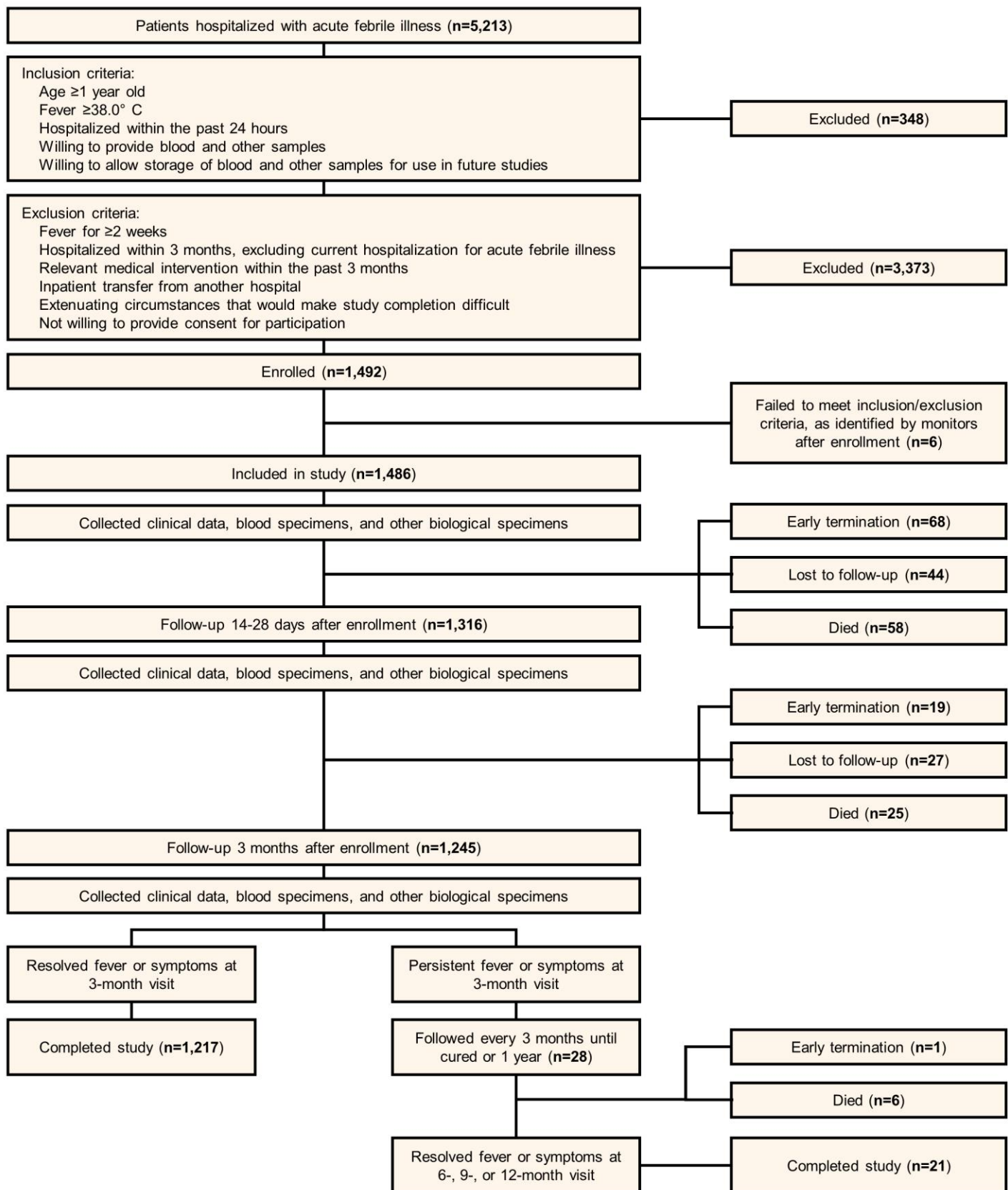

Supplement: S1 Fig — (PDF) [file pntd.0007927.s001.pdf]

**S2 Fig. Diagnostic laboratory algorithm.**

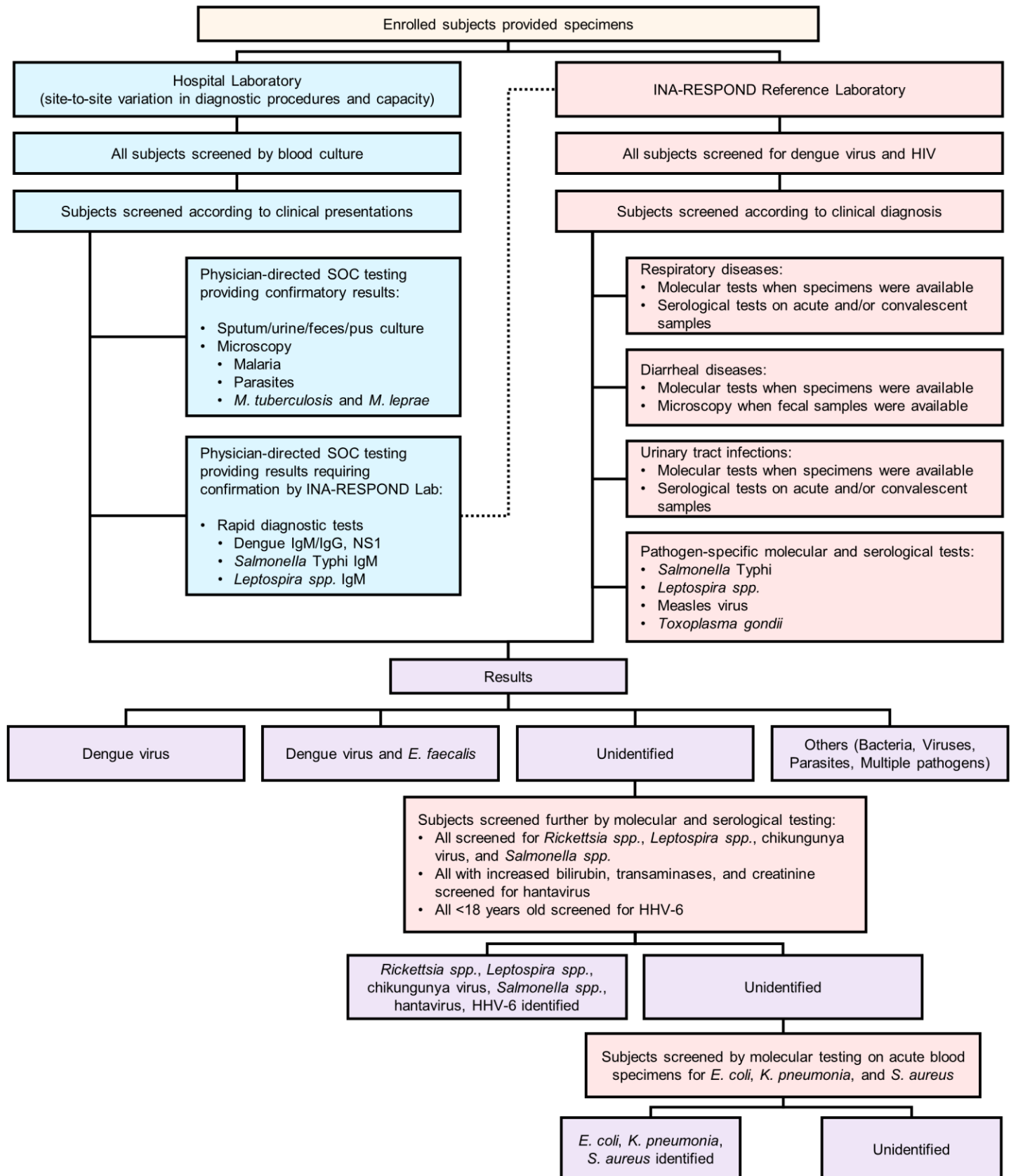

Supplement: S2 Fig — (PDF) [file pntd.0007927.s002.pdf]
