# Supplementary material for: An observational prospective cohort study of the epidemiology of hospitalized patients with acute febrile illness in Indonesia
Source: PLoS Negl Trop Dis. 2020 Jan 10;14(1):e0007927. doi: 10.1371/journal.pntd.0007927 (PMC6977771; doi:10.1371/journal.pntd.0007927)
Supplement: S1 Table — (PDF) [file pntd.0007927.s003.pdf]

S1 Table. Definitions for categorizing clinical manifestations.

Signs/Symptoms	Description
Constitutional	Fever and one or more of the following: headache, dizziness, anorexia, myalgia, arthralgia, joint pain, lethargic, paleness
Central Nervous System	Fever with decrease of consciousness or febrile convulsion
Upper Respiratory	Fever and one or more of the following: cough, coryza, tonsillitis, pharyngitis, sore throat, otitis
Lower Respiratory	Fever and one or more of the following: shortness of breath, rhonchi, pleural effusion, decreased vesicular breathing sound, hemoptysis
Gastrointestinal	Fever and one or more of the following: nausea, vomiting, epigastric pain, abdominal pain, hematemesis, melena, constipation
Hepatobiliary	Fever with icteric or "dark/brown urine" or hepatomegaly
Diarrheal	Fever with changes in feces consistency and defecation frequency (>=3)
Skin and Soft Tissues	Fever and one or more of the following: skin rash, petechiae, ecchymosis, lymphadenopathy, ulcer, cellulitis
Urinary Tract Infection / Pelvic Inflammatory Disease	Fever and one or more of the following: flank pain, suprapubic pain, dysuria
