# Supplementary material for: An observational prospective cohort study of the epidemiology of hospitalized patients with acute febrile illness in Indonesia
Source: PLoS Negl Trop Dis. 2020 Jan 10;14(1):e0007927. doi: 10.1371/journal.pntd.0007927 (PMC6977771; doi:10.1371/journal.pntd.0007927)
Supplement: S2 Table — (PDF) [file pntd.0007927.s004.pdf]

**S2 Table. Demography of the participants at all study sites.**

| **Patient characteristics** | | | | | | |
|---|---|---|---|---|---|---|
| **Sites** | **Male** | **Female** | **≤5 years** | **6-18 years** | **>18 years** | **Total** |
| **Bandung** | 136 | 133 | 38 | 79 | 152 | 269 |
| **Denpasar** | 134 | 79 | 5 | 47 | 161 | 213 |
| **Jakarta** | 87 | 69 | 41 | 58 | 57 | 156 |
| **Makassar** | 106 | 95 | 15 | 39 | 147 | 201 |
| **Semarang** | 134 | 123 | 41 | 81 | 135 | 257 |
| **Surabaya** | 134 | 87 | 18 | 67 | 136 | 221 |
| **Yogyakarta** | 99 | 70 | 52 | 42 | 75 | 169 |
| **Total** | 830 | 656 | 210 | 413 | 863 | 1,486 |
