# Supplementary material for: An observational prospective cohort study of the epidemiology of hospitalized patients with acute febrile illness in Indonesia
Source: PLoS Negl Trop Dis. 2020 Jan 10;14(1):e0007927. doi: 10.1371/journal.pntd.0007927 (PMC6977771; doi:10.1371/journal.pntd.0007927)
Supplement: S3 Table — (PDF) [file pntd.0007927.s005.pdf]

S3 Table. Molecular and serology tests at the INA-RESPOND Laboratory.

Pathogen	Methods	Confirmation
Dengue virus	Molecular: real-time PCR[1] and nested PCR[2]<br>Antigen detection (Focus Technology, US)<br>Serology assay: ELISA IgM and IgG (Focus Technology, US)	DENV RNA was detected<br>NS1 antigen was detected<br>Sero-conversion in IgM and or IgG between acute and convalescent specimens; four-fold increase of IgG in the absence of IgM; detectable IgM, supported by IgG and clinical manifestations.
Salmonella Typhi / *Salmonella paratyphi*	Blood culture (Bactec or ViTek)<br>Molecular: *S.* Typhi[3], *S. paratyphi*[4]<br>*S.* Typhi antibodies by ELISA (MyBioSource, US) IgM and IgG	Identified by API<br>*S.* Typhi/*S. paratyphi* DNA was detected<br>Sero-conversion or increasing of IgM and/or IgG antibodies titer. Detection of IgM in acute samples, supported by clinical manifestations.
Rickettsia typhi	Molecular: *Rickettsia spp.*[5], *R. typhi*[6]<br>*R. typhi* ELISA IgM/IgG antibodies using ELISA (Fuller, US)<br>Murine typhus IFA (Focus, US)	*R. typhi* DNA was detected, if only *Rickettsia spp.* is detectable, it is supported by *R. typhi* IgM and IgG antibodies<br>Sero-conversion or increasing titers of *R. typhi* IgM and IgG antibodies in convalescent specimens.<br>Increase in Fluorescein level in convalescent specimens, in line with ELISA results
Orientia tsutsugamuchi	Molecular: *Orientia tsutsugamuchi*[5]<br>Scrub typhus ELISA IgM and IgG antibodies (InBios, US)	*O. tsutsugamuchi* DNA was detected<br>Sero-conversion or increasing titers of IgM and/or IgG *O. tsutsugamuchi* antibodies in convalescent specimens
Leptospira spp.	Micro agglutination test (MAT) in a few specimens<br>Molecular: real time PCR[7]<br>ELISA IgM leptospira (PanBio, Aus), IgG Leptospira (Serion, Germany)	Four-fold increase of Leptospira antibodies to certain species, or a high titer (1/320) of antibodies in a single acute specimen<br>Leptospira DNA was detected<br>Sero-conversion or increasing IgM and IgG titers in convalescent specimens
Chikungunya virus	Molecular: real time PCR[8]<br>ELISA IgM and IgG chikungunya antibodies (Euroimmune, Germany)	Chikungunya RNA was detected<br>Sero-conversion or increasing titers of IgM and IgG in convalescent specimens
Plasmodium spp.	Thick blood smear (microscopic)<br>Rapid Antibody test (SD Bioline, US)	Plasmodium was detected<br>Antibody was detected
Mycobacterium tuberculosis	Acid fast bacilli smear (microscopic)	Acid fast bacilli was detected
Mycobacterium leprae	Acid fast bacilli smear (microscopic)	Acid fast bacilli was detected
Amoeba coli / *Amoeba histolytica*	Microscopic examination	Amoeba was detected
Helminthiasis	Microscopic examination	Helmith eggs or spores were detected
Other parasites	Microscopic examination	A parasite was detected
Seoul virus	Molecular: conventional PCR[9]<br>ELISA IgM and IgG Hantavirus Antibodies (Focus diagnostics)	Seoul virus RNA was detected<br>Sero-conversion or increasing titers of IgM and IgG Hantavirus anti bodies in convalescent specimens
Rubella virus	ELISA Rubella virus IgM and IgG antibodies (Serion, Germany)	Sero-conversion or increasing titers of IgM and IgG antibodies in convalescent specimens
Influenza virus	Molecular: multiplex real-time PCR[10]<br>ELISA Influenza A and B virus IgM and IgG antibodies (Serion, Germany)	Influenza virus RNA was detected<br>Two-fold increase of IgM and/or IgG influenza A or Influenza B antibodies in convalescent specimens
Respiratory syncytial virus	Molecular: multiplex real-time PCR[10]<br>ELISA RSV IgM and IgG antibodies (Serion, Germany)	RSV RNA was detected<br>Sero-conversion or increasing of IgM or IgG RSV antibodies in convalescent specimens
Parainfluenza viruses	Melecular: multiplex real-time PCR[10]	Para influenza virus RNA was detected
Adenovirus	Melecular: multiplex real-time PCR[10]	Adenovirus RNA was detected
Coronavirus OC43	Melecular: multiplex real-time PCR[10]	CorOC43 virus RNA was detected
Parechovirus	Melecular: multiplex real-time PCR[10]	Parechovirus RNA was detected
Bocavirus	Melecular: multiplex real-time PCR[10]	Bocavirus RNA was detected
Rhinovirus	Melecular: multiplex real-time PCR[10]	Rhinovirus RNA was detected

Pathogen	Methods	Confirmation
Metapneumovirus	Molecular: multiplex real-time PCR[10]	Metapneumovirus RNA was detected
Measles virus	Molecular: conventional[11]<br>ELISA measles virus IgM and IgG antibodies (Serion, Germany)	Measles virus RNA was detected<br>Sero-conversion or increase titers of IgM and/or IgG antibodies in convalescent specimens
HHV-6	Conventional PCR[12]<br>Real-time PCR[13]	HHV-6 virus DNA was detected<br>HHV-6 virus DNA >1,000 copies/ul
Enterovirus	Real-time PCR[14]	Enterovirus RNA was detected
Flavivirus	Conventional PCR[15]	Flavivirus RNA was detected
Zika virus	Real-time PCR[16]	Zikavirus RNA was detected
Rotavirus	Real-time PCR[17]	Rotavirus RNA was detected
Norovirus	Real-time PCR[18]	Norovirus RNA was detected
Adenovirus (ent)	Real-time PCR[17]	Adenovirus RNA was detected
Astrovirus	Real-time PCR[18]	Astrovirus RNA was detected
Chlamydia pneumonia	Real-time PCR[19]	*C. pneumonia* DNA was detected
Chlamydia psitacci	Real-time PCR[20]	*C. pistacci* DNA was detected
Streptococcus pneumonia	Real-time PCR[21]	*S. pneumoniae* DNA was detected
Haemophilus influenza	Real-time PCR[21]	*H. influenza* DNA was detected
Bordetella pertussis	Real-time PCR[22]	*B. pertussis* DNA was detected
Legionella pneumoniae	Real-time PCR[23]	*L. pneumoniae* DNA was detected
Mycoplasma pneumoniae	Real-time PCR[24]	*M. pneumoniae* DNA was detected
Staphylococcus aureus	Real-time PCR[25]	*S. aureus* DNA was detected
Klebsiella pneumoniae	Real-time PCR[25]	*K. pneumoniae* DNA was detected
16S r RNA	Real-time PCR[7]	Bacterial DNA was detected
HIV	Real-time PCR[26]<br>Rapid Test SD®, Oncoprobe®<br>ELISA 4th generation (Biorad®)	HIV RNA was detected<br>HIV antibody was negative<br>4th generation ELISA was positive

Footnotes:

1. Hue KD, Tuan TV, Thi HT, et al. Validation of an internally controlled one-step real-time multiplex RT-PCR assay for the detection and quantitation of dengue virus RNA in plasma. *J Virol Methods* 2011; **177**(2):168–73.
2. Lanciotti RS, Calisher CH, Gubler DJ, Chang GJ, Vorndam AV. Rapid detection and typing of dengue viruses from clinical samples by using reverse transcriptase-polymerase chain reaction. J Clin Microbiol 1992; 30(3):545–51.
3. Hatta M, Smits HL. Detection of Salmonella typhi by nested polymerase chain reaction in blood, urine, and stool samples. Am J Med Trop Med Hyg 2007; 76(1):139–43.
4. Pratap CB, Kumar G, Patel SK, et al. Mix-infection of S. typhi and paratyphi A in typhoid fever and chronic typhoid carriers: a nested PCR based study in North India. J Clin Diagn Res 2014; 8(11):DC09–DC14.
5. Jiang J, Chan TC, Temenak JJ, Dasch GA, Ching WM, Richards AL. Development of a quantitative real-time polymerase chain reaction assay specific for Orientia tsutsugamushi. Am J Med Trop Med Hyg 2004; 70(4):351–6.
6. Henry KM, Jiang J, Rozmajzl PJ, Azad AF, Macaluso KR, Richards AL. Development of quantitative real-time PCR assays to detect Rickettsia typhi and Rickettsia felis, the causative agents of murine typhus and flea-borne spotted fever. Mol Cell Probes 2007; 21(1):17–23.
7. Thaipadungpanit J, Chierakul W, Wuthiekanun V, et al. Diagnostic accuracy of real-time PCR assays targeting 16S rRNA and lipL32 genes for human leptospirosis in Thailand: a case-control study. PLoS One 2011; 6(1).
8. Lanciotti RS, Kosoy OL, Laven JJ, et al. Chikungunya virus in US travelers returning from India, 2006. Emerg Infect Dis 2007; 13(5):764–7.
9. Dekonenko A, Ibrahim MS, Schmaljohn CS. A colorimetric PCR-enzyme immunoassay to identify hantaviruses. Clin Diagn Virol 1997; 8(2):113–21.
10. Jansen RR, Schinkel J, Koekkoek S, et al. Development and evaluation of a four-tube real time multiplex PCR assay covering fourteen respiratory viruses, and comparison to its corresponding single target counterparts. Clin Diagn Virol 2011; 51(3):179–85.

11. Chibo D, Birch CJ, Rota PA, Catton MG. Molecular characterization of measles viruses isolated in Victoria, Australia, between 1973 and 1998. J Gen Virol 2000; 81(Pt 10):2511–8.
12. Huang LM, Kuo PF, Lee CY, Chen JY, Liu MY, Yang CS. Detection of human herpesvirus-6 DNA by polymerase chain reaction in serum or plasma. J Med Virol 1992; 38(1):7–10.
13. Sedlak RH, Cook L, Huang ML, et al. Identification of chromosomally integrated human herpesvirus 6 by droplet digital PCR. Clin Chem 2014; 60(5):765–72.
14. Beld M, Minnaar R, Weel J, et al. Highly sensitive assay for detection of enterovirus in clinical specimens by reverse transcription-PCR with an armored RNA internal control. J Clin Microbiol 2004; 42(7):3059–64.
15. Kuno G, Chang G-JJ, Tsuchiya KR, Karabatsos N, Cropp CB. Phylogeny of the genus Flavivirus. J Virol 1998; 72(1):73–83.
16. Lanciotti RS, Kosoy OL, Laven JJ, et al. Genetic and serologic properties of zika virus associated with an epidemic, Yap State, Micronesia, 2007. Emerg Infect Dis 2008; 14(8).
17. Logan C, O'Leary JJ, O'Sullivan N. Real-time reverse transcription-PCR for detection of rotavirus and adenovirus as causative agents of acute viral gastroenteritis in children. J Clin Microbiol 2006; 44(9):3189–95
18. Logan C, O'Leary JJ, O'Sullivan N. Real-time reverse transcription PCR detection of norovirus, sapovirus and astrovirus as causative agents of acute viral gastroenteritis. J Virol Methods 2007; 146(1-2):36–44.
19. Heddema ER PY, Langerak AA, Beld M, Duim B. Development of an internally controlled Taqman based PCR assay for the detection of Chlamydia pneumoniae in the Lightcycler 2.0 system. Ned Tijschr Med Microbiol 2004; 12(s1:s61).
20. Heddema ER, Beld MGHM, Wever Bd, Langerak AAJ, Pannekoek Y, Duim B. Development of an internally controlled real-time PCR assay for detection of Chlamydophila psittaci in the LightCycler 2.0 system. Clin Microbiol Infect 2006; 12(6):571–5.
21. Corless CE, Guiver M, Borrow R, Edwards-Jones V, Fox AJ, Kaczmarski EB. Simultaneous Detection of Neisseria meningitidis, Haemophilus influenzae, and Streptococcus pneumoniae in suspected cases of meningitis and septicemia using real-time PCR. J Clin Microbiol 2001; 39(4):1553–8
22. Reischl U, Lehn N, Sanden GN, Loeffelholz MJ. Real-time PCR assay targeting IS481 of Bordetella pertussis and molecular basis for detecting Bordetella holmesii. J Clin Microbiol 2001; 39(5):1963–6.
23. Wilson DA, Yen-Lieberman B, Reischl U, Gordon SM, Procop GW. Detection of Legionella pneumophila by real-time PCR for the mip gene. J Clin Microbiol 2003; 41(7):3327–30.
24. Pitcher D, Chalker VJ, Sheppard C, George RC, Harrison TG. Real-time detection of Mycoplasma pneumoniae in respiratory samples with an internal processing control. J Med Microbiol 2006; 55:149–55.
25. Gadsby NJ, McHugh MP, Russell CD, Mark H, Conway Morris A, Laurenson IF, Hill AT, Templeton KE. 2015. Development of two real-time multiplex PCR assays for the detection and quantification of eight key bacterial pathogens in lower respiratory tract infections. Clin Microbiol Infect 2015; 21: 788.e1–788.e13
26. Palmer S, Wiegand AP, Maldarelli F, et al. New real-time reverse transcriptase-initiated PCR assay with single-copy sensitivity for Human Immunodeficiency Virus type 1 RNA in plasma. J Clin Microbiol 2003; 41(10):4531–6
