# Supplementary material for: An observational prospective cohort study of the epidemiology of hospitalized patients with acute febrile illness in Indonesia
Source: PLoS Negl Trop Dis. 2020 Jan 10;14(1):e0007927. doi: 10.1371/journal.pntd.0007927 (PMC6977771; doi:10.1371/journal.pntd.0007927)
Supplement: S4 Table — (PDF) [file pntd.0007927.s006.pdf]

S4 Table. Diagnostic discrepancies between clinical or hospital SOC testing and INA-RESPOND Laboratory diagnosis.

All Etiologies (n=1,003)	Diagnosed by INA-RESPOND Lab only (n=351)	Clinical diagnosis by hospital at discharge
**Dengue virus (467)**	44 (9.4%)	Typhoid fever (16), Fever (7), URTI (3), UTI (3), Viral infection (4), CAP (3), Acute leukemia, Gastroenteritis, Encephalitis, Colitis TB, Lung TB, Metabolic encephalopathy, Febrile convulsion, Morbili*
**Rickettsia (103)**	103 (100%)	Typhoid fever (42), Dengue (21), Fever (6), Leptospirosis (6), Sepsis (5), CAP (5), Viral infection (3), UTI (3), URTI (1), HIV, Neuropathic diabetic, Pancreatitis, Bronchitis, Blood stream infection, Chikungunya, Cholecystitis, Dysentery, Hepatitis, Meningoencephalitis, Paratyphoid fever*
***Salmonella enterica* (103)**	16 (16%)	Dengue (5), Gastroenteritis (3), CAP, Hirschsprung's disease, HIV, UTI, Aseptic meningitis, Fever, URTI, Viral infection*
**Influenza (68)**	68 (100%)	URTI (18), CAP (19), Typhoid fever (9), Bronchiectasis (3), Lung TB (6), Dengue (5), Fever (2) Blood stream infection, Febrile convulsion, UTI, Sepsis, Gastroenteritis, Tendonitis*
**Leptospira (44)**	25 (57%)	Dengue (10), UTI (3), Sepsis (2), Typhoid fever (3), CAP (2), Gastroenteritis (2), Fever, URTI, Diabetic ulcer*
**Chikungunya (37)**	37 (100%)	Dengue (11), Typhoid fever (7), Fever (3), Viral infection (3), Febrile convulsion (3), URTI (2), COPD, Gastroenteritis, CAP, Cellulitis, Exanthema subitum, HSV-1, Leptospirosis, Rejected infective endocarditis*
***Escherichia coli* (21)**	0	
***Mycobacterium tuberculosis* (20)**	0	
***Streptococcus pneumoniae* (20)**	18 (90%)	CAP (7), Typhoid fever (2), Sepsis (2), Lung TB, Diabetic ulcer, Gastroenteritis, Fever, Febrile convulsion, Lupus nephritis, Thyroiditis*
**Measles (14)**	3 (21%)	Dengue (2), Typhoid fever (1)
**Amoeba (11)**	2 (18%)	Febrile convulsion (1), Typhoid fever (1)
**RSV (11)**	11 (100%)	CAP (7), URTI (2), Bronchiolitis, Pleuritis TB*
**Other (79):**	**21 (26.6%):**	
Klebsiella pneumoniae (13)	1 (7.6%)	Hepatic abscess*
HHV-6 (9)	9 (100%)	Viral infection (2), URTI (2), CMV, Paralytic ileus, CAP, Lung TB, Sepsis*
Staphylococcus aureus (8)	0	
Pseudomonas aeruginosa (8)	0	
Acinetobacter baumanii (7)	1 (14.3%)	Typhoid fever*
Hepatitis A (6)	0	
Enterococcus faecalis (4)	0	
Enterobacter aerogenes (2)	0	
Malaria (2)	0	

All Etiologies (n=1,003)	Diagnosed by INA-RESPOND Lab only (n=351)	Clinical diagnosis by hospital at discharge
Mycobacterium leprae (2)	0	
Seoul virus (2)	2 (100%)	Typhoid fever, Dengue*
Adenovirus (1)	1 (100%)	URTI*
Ascaris lumbricoides (1)	1 (100%)	Typhoid fever*
Enterobacter cloacae (1)	0	
Enterococcus avium (1)	0	
Enterovirus (1)	1 (100%)	Acute otitis media
Human coronavirus oc43 (1)	1 (100%)	URTI
HIV (1)	1 (100%)	CAP
Meta pneumovirus (1)	1 (100%)	CAP
Mycoplasma pneumonia (1)	1 (100%)	CAP
Norovirus II (1)	1 (100%)	Gastroenteritis
Pseudomonas cepacea (1)	0	
Rubella (1)	0	
Staphylococcus haemolyticus (1)	0	
Streptococcus viridans (1)	0	
Streptococcus faecalis (1)	0	
Streptococcus pyogenes (1)	0	
**Multiple (5):**	**3 (29%):**	
Ascaris lumbricoides, Trichuris trichiura (1)	1 (100%)	Urticaria
Bordetella pertussis, Streptococcus pneumoniae (1)	1 (100%)	Bronchiectasis
Dengue, *Enterococcus faecalis* (1)	1 (100%)	UTI
Enterococcus- and Staphylococcus aureus (1)	0	
*Moraxella catarrhalis and Influenza B** (1)*	0	

* 1 case for each diagnosis
** Influenza B confirmed by INA-RESPOND Lab
† 5 patients were infected with multiple pathogens from the above table and are not shown to avoid duplication
