# Supplementary material for: An observational prospective cohort study of the epidemiology of hospitalized patients with acute febrile illness in Indonesia
Source: PLoS Negl Trop Dis. 2020 Jan 10;14(1):e0007927. doi: 10.1371/journal.pntd.0007927 (PMC6977771; doi:10.1371/journal.pntd.0007927)
Supplement: S5 Table — (PDF) [file pntd.0007927.s007.pdf]

S5 Table. List of pathogens according to clinical syndromes.

One Syndrome	Pathogens			
	Viruses	Bacteria	Parasites	Multiple Pathogens
Constitutional (62)				
Unknown (21)				
Identified (41)	Chikungunya (2)<br>Dengue (27)	*Rickettsia typhi* (6)<br>*Salmonella paratyphi* A (2)<br>*Salmonella* Typhi (3)<br>*Streptococcus viridans* (1)		
Central nervous system (17)				
Unknown (8)				
Identified (9)	Chikungunya (1)<br>Dengue (4)<br>HHV-6 (1)<br>Influenza (1)	*Salmonella spp.* (1)<br>*Streptococcus pneumoniae* (1)		
Upper respiratory tract (39)				
Unknown (17)				
Identified (22)	Dengue (7)<br>Adenovirus (1)<br>Influenza (4)<br>Chikungunya (1)	*Escherichia coli* (1)<br>*Acinetobacter baumanii* (1)<br>*Mycobacterium tuberculosis* (2)<br>*Leptospira spp.* (1)<br>*Salmonella* Typhi (3)		*Bordetella pertussis* and *Streptococcus pneumoniae* (1)
Lower respiratory (75)				
Unknown (32)				
Identified (43)	HHV-6 (1)<br>Chikungunya (1)<br>Influenza (10)<br>Measles virus (1)<br>Metapneumovirus (1)<br>RSV (7)	*Acinetobacter baumanii* (3)<br>*Enterobacter aerogenes* (1)<br>*Escherichia coli* (2)<br>*Klebsiella pneumoniae* (2)<br>*Mycobacterium tuberculosis* (5)<br>*Pseudomonas aeruginosa* (2)<br>*Salmonella* Typhi (1)<br>*Rickettsia typhi* (1)<br>*Streptococcus pneumoniae* (1)<br>*Staphylococcus aureus* (3)<br>*Enterobacter cloacae* (1)		
Urinary tract infection (4)				
Unknown (3)				
Identified (1)	Dengue (1)			
Gastrointestinal (497)				
Hepatobiliary (28)				
Unknown (10)				
Identified (18)	Dengue (2)<br>Hepatitis A (5)	*Enterobacter aerogenes* (1)<br>*Escherichia coli* (1)<br>*Leptospira spp.* (4)<br>*Rickettsia felis* (1)<br>*Rickettsia typhi* (2)<br>*Salmonella* Typhi (1)	Malaria (1)	

One Syndrome	Pathogens			
	Viruses	Bacteria	Parasites	Multiple Pathogens
Diarrheal disease (104)				
Unknown (43)				
Identified (61)	Chikungunya (1)	*Escherichia coli* (3)	Amoeba (4)	
	Dengue (21)	*Leptospira spp.* (8)	*Entamoeba coli* (1)	
	Norovirus II (1)	*Rickettsia typhi* (7)	*Entamoeba histolytica* (1)	
		Salmonella spp. (1)		
		Salmonella Typhi (12)		
Non-diarrheal disease (365)				
Unknown (66)				
Identified (299)	Chikungunya (9)	*Acinetobacter baumanii* (1)	Malaria (1)	
	Dengue (203)	*Pseudomonas aeruginosa* (1)		
	HHV-6 (1)	*Escherichia coli* (5)		
	Hepatitis A (1)	*Klebsiella pneumoniae* (3)		
	Influenza (4)	*Leptospira spp.* (7)		
	Seoul virus (1)	*Mycobacterium tuberculosis* (1)		
		Rickettsia typhi (38)		
		Salmonella paratyphi A (1)		
		Salmonella Typhi (22)		
Skin and soft tissue (24)				
Rash and bleeding (16)				
Unknown (4)				
Identified (12)	Chikungunya (1)	*Acinetobacter baumanii* (1)		
	Dengue (8)	*Rickettsia typhi* (2)		
Other (8)				
Unknown (0)				
Identified (8)	HHV-6 (1)	*Enterococcus avium* (1)		*Ascaris lumbricoides* and *Trichuris trichiura* (1)
		Enterococcus faecalis (1)		
		Mycobacterium leprae (1)		
		Rickettsia typhi (1)		
		Staphylococcus aureus (2)		

Two Syndromes	Pathogens			
	**Viruses**	**Bacteria**	**Parasites**	**Multiple Pathogens**
Lower respiratory and gastrointestinal (71)				
Unknown (33)				
Identified (38)	Chikungunya (2)<br>Dengue (5)<br>Influenza (9)<br>RSV (1)<br>HIV (1)	*Acinetobacter baumanii* (1)<br>*Leptospira spp.* (1)<br>*Mycobacterium tuberculosis* (7)<br>*Pseudomonas aeruginosa* (2)<br>*Rickettsia typhi* (2)<br>*Salmonella* Typhi (2)<br>*Streptococcus pneumoniae* (2)<br>*Klebsiella pneumoniae* (2)		*Moraxella catarrhalis* and Influenza B (1)
Lower respiratory and diarrheal disease (35)				
Unknown (15)				
Identified (20)	Dengue (2)<br>Influenza (2)	*Leptospira spp.* (3)<br>*Mycobacterium tuberculosis* (3)<br>*Pseudomonas aeruginosa* (1)<br>*Rickettsia typhi* (1)<br>*Salmonella* Typhi (4)<br>*Streptococcus pneumoniae* (4)		
Lower respiratory and urinary tract infection (3)				
Unknown (1)				
Identified (2)		*Pseudomonas cepacea* (1)<br>*Mycoplasma pneumonia* (1)		
Lower respiratory and hepatobiliary (3)				
Unknown (1)				
Identified (2)		*Leptospira spp.* (1)<br>*Streptococcus pneumonia* (1)		
Central nervous system and upper respiratory (22)				
Unknown (11)				
Identified (11)	Chikungunya (3)<br>Dengue (3)<br>Influenza (2)<br>RSV (1)<br>HHV-6 (1)	*Enterococcus faecalis* (1)		
Central nervous system and diarrheal disease (10)				
Unknown (9)				
Identified (1)			Amoeba (1)	
Central nervous system and gastrointestinal (9)				
Unknown (3)				
Identified (6)	Dengue (3)	*Leptospira spp.* (1)<br>*Rickettsia typhi* (2)		
Central nervous system and hepatobiliary (1)				
Unknown (0)				
Identified (1)	Seoul virus (1)			
Central nervous system and lower respiratory (12)				
Unknown (6)				
Identified (6)	Influenza (2)<br>RSV (1)	*Escherichia coli* (1)<br>*Klebsiella pneumoniae* (1)<br>*Streptococcus pneumoniae* (1)		

Two Syndromes	Pathogens			
	Viruses	Bacteria	Parasites	Multiple Pathogens
Urinary tract infection and diarrheal disease (9)				
Unknown (4)				
Identified (5)	Dengue (2)	*Leptospira spp.* (1)		
		Salmonella Typhi (2)		
Urinary tract infection and hepatobiliary (1)				
Unknown (0)				
Identified (1)	Dengue (1)			
Upper respiratory and diarrheal disease (59)				
Unknown (22)				
Identified (37)	Coronavirus OC43 (1)	*Escherichia coli* (2)	*Entamoeba histolytica* (1)	
	Dengue (10)	*Leptospira spp.* (4)		
	Enterovirus (1)	*Pseudomonas aeruginosa* (1)		
	Influenza (1)	*Rickettsia typhi* (2)		
		Salmonella Typhi (14)		
Upper respiratory and gastrointestinal (183)				
Unknown (64)				
Identified (119)	Dengue (44)	*Escherichia coli* (1)		
	Chikungunya (6)	*Klebsiella pneumoniae* (2)		
	Influenza (26)	*Leptospira spp.* (3)		
	RSV (1)	*Mycobacterium tuberculosis* (2)		
		Rickettsia typhi (11)		
		Salmonella paratyphi A (4)		
		Salmonella spp. (1)		
		Salmonella Typhi (15)		
		Staphylococcus aureus (1)		
		Streptococcus pneumoniae (2)		
Upper respiratory and hepatobiliary (9)				
Unknown (3)				
Identified (6)	Dengue (1)	*Leptospira spp.* (2)		
	HHV-6 (1)	*Rickettsia typhi* (2)		
Upper respiratory and skin & soft tissue (36)				
Unknown (16)				
Identified (20)	Dengue (5)	*Escherichia coli* (2)		Dengue and *Enterococcus faecalis* (1)
	Influenza (1)	*Leptospira spp.* (1)		
	Measles (3)	*Rickettsia typhi* (2)		
		Salmonella paratyphi A (2)		
		Staphylococcus aureus (1)		
		Pseudomonas aeruginosa (1)		
		Klebsiella pneumonia (1)		
Lower respiratory and skin & soft tissue (rash and bleeding) (4)				
Unknown (1)				
Identified (3)	Measles (2)			
	Dengue (1)			
Lower respiratory and skin & soft tissue (without rash and bleeding) (1)				
Unknown (0)				
Identified (1)		*Salmonella spp.* (1)		

Two Syndromes	Pathogens			
	Viruses	Bacteria	Parasites	Multiple Pathogens
Diarrheal disease and skin & soft tissue (rash and bleeding) (13)				
Unknown (1)				
Identified (12)	Dengue (8)	*Leptospira spp.* (1)<br>*Rickettsia typhi* (2)<br>*Salmonella* Typhi (1)		
Diarrheal disease and skin & soft tissue (without rash and bleeding) (3)				
Unknown (1)				
Identified (2)		*Escherichia coli* (1)<br>*Salmonella* Typhi (1)		
Gastrointestinal and skin & soft tissue (rash and bleeding) (100)				
Unknown (18)				
Identified (82)	Chikungunya (3)<br>Dengue (67)<br>Influenza (1)	*Rickettsia typhi* (9)<br>*Salmonella paratyphi* A (1)<br>*Streptococcus pneumoniae* (1)		
Gastrointestinal and skin & soft tissue (without rash and bleeding) (9)				
Unknown (3)				
Identified (6)		*Enterococcus faecalis* (1)<br>*Mycobacterium leprae* (1)<br>*Salmonella spp.* (1)<br>*Streptococcus pneumoniae* (1)<br>*Streptococcus faecalis* (1)		*Enterococcus* and *Staphylococcus aureus* (1)

Three Syndromes	Pathogens			
	**Viruses**	**Bacteria**	**Parasites**	**Multiple Pathogens**
Upper respiratory, gastrointestinal, skin & soft tissue (rash and bleeding) (7)				
Unknown (4)				
Identified (3)	Dengue (1)<br>Chikungunya (1)<br>Measles (1)			
Upper respiratory, gastrointestinal, skin & soft tissue (without rash and bleeding) (50)				
Unknown (9)				
Identified (41)	Chikungunya (1)<br>Dengue (27)<br>Measles (2)	*Rickettsia typhi* (6)<br>*Salmonella* Typhi (1)<br>*Staphylococcus haemolyticus* (1)<br>*Klebsiella pneumoniae* (1)<br>*Streptococcus pyogenes* (1)<br>*Leptospira spp.* (1)		
Upper respiratory, diarrheal disease, skin & soft tissue (rash and bleeding) (5)				
Unknown (1)				
Identified (4)	Measles (3)<br>Rubella (1)			
Upper respiratory, diarrheal disease, skin & soft tissue (without rash and bleeding) (9)				
Unknown (2)				
Identified (7)	Dengue (3)<br>HHV-6 (1)<br>Measles (1)	*Salmonella* Typhi (1)	*Entamoeba coli* (1)	
Central nervous system, upper respiratory, diarrheal disease (13)				
Unknown (8)				
Identified (5)		*Enterococcus faecalis* (1)<br>*Salmonella* Typhi (3)	Amoeba (1)	
Central nervous system, upper respiratory, gastrointestinal (12)				
Unknown (9)				
Identified (3)	Chikungunya (1)<br>HHV-6 (1)<br>Influenza (1)			
Central nervous system, diarrheal disease, skin & soft tissue (rash and bleeding) (1)				
Unknown (0)				
Identified (1)		*Rickettsia typhi* (1)		
Central nervous system, gastrointestinal, skin & soft tissue (without rash and bleeding) (1)				
Unknown (0)				
Identified (1)	Dengue (1)			

Three Syndromes	Pathogens			
	Viruses	Bacteria	Parasites	Multiple Pathogens
Upper respiratory, urinary tract infection, gastrointestinal (10)				
Unknown (5)				
Identified (5)	Chikungunya (1)	*Rickettsia typhi* (3)<br>*Streptococcus pneumoniae* (1)		
Lower respiratory, gastrointestinal, skin & soft tissue (rash and bleeding) (2)				
Unknown (1)				
Identified (1)		*Streptococcus pneumoniae* (1)		
Lower respiratory, gastrointestinal, skin & soft tissue (without rash and bleeding) (6)				
Unknown (3)				
Identified (3)	Dengue (2)<br>Influenza (1)			
Lower respiratory, gastrointestinal, urinary tract infection (8)				
Unknown (5)				
Identified (3)	Influenza (1)	*Escherichia coli* (1)<br>*Streptococcus pneumoniae* (1)		
Gastrointestinal, urinary tract infection, skin & soft tissue (rash and bleeding) (2)				
Unknown (1)				
Identified (1)	Chikungunya (1)			
Gastrointestinal, urinary tract infection, skin & soft tissue (without rash and bleeding) (6)				
Unknown (1)				
Identified (5)	Dengue (3)	*Rickettsia typhi* (1)<br>*Salmonella* Typhi (1)		
Central nervous system, lower respiratory, gastrointestinal (6)				
Unknown (3)				
Identified (3)	Dengue (1)<br>Influenza (1)	*Staphylococcus aureus* (1)		
Central nervous system, lower respiratory, diarrheal disease (4)				
Unknown (2)				
Identified (2)	Chikungunya (1)	*Rickettsia typhi* (1)		
Central nervous system, lower respiratory, skin & soft tissue (1)				
Unknown (1)				
Identified (0)				
Lower respiratory, diarrheal disease, skin & soft tissue (rash and bleeding) (5)				
Unknown (2)				
Identified (3)	Chikungunya (1)<br>Measles (1)	*Leptospira spp.* (1)		

Three Syndromes	Pathogens			
	Viruses	Bacteria	Parasites	Multiple Pathogens
Lower respiratory, diarrheal disease, skin & soft tissue (without rash and bleeding) (1)				
Unknown (0)				
Identified (1)		*Streptococcus pneumoniae* (1)		
Upper respiratory, hepatobiliary, skin & soft tissue (rash and bleeding) (3)				
Unknown (2)				
Identified (1)		*Leptospira spp.* (1)		
Upper respiratory, hepatobiliary, skin & soft tissue (without rash and bleeding) (1)				
Unknown (0)				
Identified (1)		*Leptospira spp.* (1)		
Upper respiratory, urinary tract infection, diarrheal disease (3)				
Unknown (1)				
Identified (2)		*Salmonella* Typhi (1)	*Entamoeba coli* (1)	
Lower respiratory, hepatobiliary, skin & soft tissue (rash and bleeding) (2)				
Unknown (1)				
Identified (1)		*Streptococcus pneumoniae* (1)		
Upper respiratory, hepatobiliary, urinary tract infection (1)				
Unknown (1)				
Identified (0)				
Lower respiratory, hepatobiliary, urinary tract infection (2)				
Unknown (0)				
Identified (2)		*Leptospira spp.* (2)		
Central nervous system, lower respiratory, skin & soft tissue (without rash and bleeding) (1)				
Unknown (1)				
Identified (0)				
Urinary tract infection, hepatobiliary, skin & soft tissue (without rash and bleeding) (1)				
Unknown (0)				
Identified (1)			*Ascaris lumbricoides* (1)	
Lower respiratory, diarrheal disease, urinary tract infection (1)				
Unknown (0)				
Identified (1)		*Escherichia coli* (1)		

Four Syndromes	Pathogens			
	Viruses	Bacteria	Parasites	Multiple Pathogens
Central nervous system, lower respiratory, gastrointestinal, skin & soft tissue (without rash and bleeding) (2)				
Unknown (1)				
Identified (1)	Dengue (1)			
Central nervous system, upper respiratory, diarrheal disease, skin & soft tissue (rash and bleeding) (1)				
Unknown (0)				
Identified (1)		*Klebsiella pneumoniae* (1)		
Central nervous system, upper respiratory, gastrointestinal, skin & soft tissue (rash and bleeding) (1)				
Unknown (0)				
Identified (1)	HHV-6 (1)			
Upper respiratory, urinary tract infection, diarrheal disease, skin & soft tissue (rash and bleeding) (2)				
Unknown (1)				
Identified (1)	Dengue (1)			
Upper respiratory, urinary tract infection, gastrointestinal, skin & soft tissue (rash and bleeding) (2)				
Unknown (0)				
Identified (2)	Dengue (1)<br>Influenza (1)			
Central nervous system, upper respiratory, urinary tract infection, gastrointestinal (1)				
Unknown (0)				
Identified (1)	Dengue (1)			
Central nervous system, upper respiratory, urinary tract infection, diarrheal disease (1)				
Unknown (1)				
Identified (0)				
