# Supplementary material for: An observational prospective cohort study of the epidemiology of hospitalized patients with acute febrile illness in Indonesia
Source: PLoS Negl Trop Dis. 2020 Jan 10;14(1):e0007927. doi: 10.1371/journal.pntd.0007927 (PMC6977771; doi:10.1371/journal.pntd.0007927)
Supplement: S6 Table — (PDF) [file pntd.0007927.s008.pdf]

S6 Table. Methods of pathogen confirmation.

Pathogens (1,003)	Diagnostic methods
**1. Single pathogen (998)**	
**A. Viruses (621)**	
Dengue virus (467)	Plasma PCR, NS1, serology: 167<br>Plasma PCR and serology: 163<br>Plasma PCR and NS1: 29<br>NS1 and serology: 32<br>Plasma PCR: 38<br>NS1: 3<br>Serology: 35
Chikungunya virus (37)	Plasma PCR and serology: 30<br>Serology: 7
HHV-6 (9)	Quantitative PCR: 9
Influenza virus (68)	Plasma PCR and serology: 7<br>Serology: 61
Adenovirus (1)	Plasma PCR: 1
RSV (11)	Plasma PCR and serology: 1<br>Plasma PCR: 1<br>Serology: 9
Rubella (1)	Serology: 1
Coronavirus Co43 (1)	Plasma PCR: 1
Measles (14)	Plasma PCR and serology: 8<br>Plasma PCR: 1<br>Serology: 5
Metapneumovirus (1)	Swab PCR: 1
Norovirus 2 (1)	Plasma PCR: 1
Enterovirus (1)	Plasma PCR: 1
Seoul virus (2)	Plasma PCR, Serology: 2
Hepatitis A (6)	Serology: 6
HIV (1)	Plasma PCR: 1
**B. Bacteria (363)**	
Acinetobacter baumanii (7)	Resp. specimen culture: 5<br>Urine culture: 1<br>Blood 16S rRNA PCR and sequencing: 1
Enterobacter aerogenes (2)	Resp. specimen culture: 1<br>Blood culture: 1
Enterococcus avium (1)	Pus culture: 1
Enterococcus faecalis (4)	Pus culture: 2<br>Blood culture: 1<br>Urine culture: 1

Pathogens (1,003)	Diagnostic methods
Escherichia coli (21)	Blood culture: 13<br>Feces culture: 1<br>Pus culture: 1<br>Urine culture: 4<br>Plasma PCR: 1
Klebsiella pneumonia (13)	Blood culture: 5<br>Resp. specimen culture: 6<br>Resp. specimen culture, Resp. specimen PCR: 1<br>Blood 16s rRNA PCR and sequencing: 1
Leptospira spp. (44)	Plasma PCR, serology: 26<br>Plama PCR: 5<br>Serology: 13
Mycobacterium leprae (2)	Skin microscopic: 2
Mycobacterium tuberculosis (20)	Resp. specimen microscopic: 14<br>Resp. specimen microscopic and GeneXpert: 3<br>GeneXpert: 2<br>Feces microscopy: 1
Pseudomonas aeruginosa (8)	Resp. specimen culture: 4<br>Urine culture: 2<br>Blood culture: 2
Rickettsia typhi (102)	Plasma PCR and Serology: 63<br>Plasma PCR: 3<br>Serology: 36
Rickettsia felis (1)	Plasma PCR and serology: 1
Salmonella enterica (103)	Blood culture and Serology: 37<br>Blood culture and PCR and Serology: 7<br>Blood culture: 6<br>Blood culture and PCR: 1<br>Serology: 49<br>Plasma PCR and serology: 3
Staphylococcus aureus (8)	Blood culture: 5<br>Pus culture: 3
Staphylococcus haemolyticus (1)	Blood culture: 1
Streptococcus pneumoniae (20)	Blood culture: 2<br>Blood PCR: 10<br>Resp. specimen PCR: 8
Streptococcus viridans (1)	Blood culture: 1
Enterobacter cloacae (1)	Resp. specimen culture: 1
Mycoplasma pneumoniae (1)	Resp. Specimen PCR: 1
Pseudomonas cepacea (1)	Blood culture: 1
Streptococcus faecalis (1)	Urine culture: 1
Streptococcus pyogenes (1)	Blood culture: 1

Pathogens (1,003)	Diagnostic methods
**C. Parasites (14)**	
Amoeba (6)	Feces microscopy: 6
Entamoeba coli (3)	Feces microscopy: 3
Entamoeba histolytica (2)	Feces microscopy: 2
Ascaris lumbricoides (1)	Feces microscopy: 1
Malaria (2)	Blood smear: 1<br>Serology: 1
**2. Multiple pathogens (5):**	
**A. Virus and Bacteria (2)**	
Dengue, *Enterococcus faecalis (1)*	Plasma PCR, Serology; Urine culture
Influenza B, *Moraxella catarrhalis (1)*	Resp. specimen PCR, Serology; Resp. specimen culture
**B. Bacteria and Bacteria (2)**	
Bordetella pertussis, Streptococcus pneumonia (1)	Resp. specimen PCR; Resp. specimen PCR
Enterococcus, Staphylococcus aureus (1)	Urine culture; Blood culture
**C. Parasite and Parasite (1)**	
Ascaris lumbricoides, Trichuris trichiura (1)	Feces microscopy; Feces microscopy
