# Supplementary material for: An observational prospective cohort study of the epidemiology of hospitalized patients with acute febrile illness in Indonesia
Source: PLoS Negl Trop Dis. 2020 Jan 10;14(1):e0007927. doi: 10.1371/journal.pntd.0007927 (PMC6977771; doi:10.1371/journal.pntd.0007927)
Supplement: S7 Table — (PDF) [file pntd.0007927.s009.pdf]

S7 Table. Characteristics of deceased participants.

Characteristics	(n=1,486)
Patient mortality	
Mortality, n (%)	89 (5.9%)
Median age (range, IQR)	42.5 (1.1-78.9, 34.9)
Underlying conditions, n=86, n (%)	69 (80.2%)
Age group	
≤5 years, n=210, n (%)	8 (3.8%)
5-18 years, n=413, n (%)	9 (2.2%)
18-25 years, n=263, n (%)	6 (2.3%)
25-45 years, n=327, n (%)	25 (7.6%)
45-65 years, n=203, n (%)	31 (15.3%)
≥65 years, n=70, n (%)	10 (14.3%)
ICU admission, n (%)	10 (11.2%)
Median days in ICU (range)	5 (1-34)
Self-reported antibiotic use prior to hospitalization	
Yes, n (%)	6 (6.7%)
No, n (%)	64 (71.9%)
Unknown, n (%)	19 (21.3%)
Cause of death	
Septic Shock, n (%)	30 (33.7%)
Respiratory Failure, n (%)	17 (19.1%)
Other, n (%)	13 (14.6%)
Unknown, n (%)	29 (32.6%)
Laboratory-confirmed etiology, n=89, n (%)	45 (50.6%)
M. tuberculosis	8
R. typhi	7
Salmonella spp.	5
S. pneumoniae	3
E. coli	3
Dengue virus	3
Influenza virus	3
K. pneumoniae	2
A. baumanii	1
A. lumbricoides	1
E. aerogenes	1
E. avium	1
Enterococcus spp., S. aureus	1
M. catarrhalis, Influenza virus	1
Leptospira spp.	1
P. aeruginosa	1
S. aureus	1
RSV	1
HIV	1
